# Successional action of *Bacteroidota* and *Firmicutes* in decomposing straw polymers in a paddy soil

**DOI:** 10.1186/s40793-023-00533-6

**Published:** 2023-10-14

**Authors:** Junjie Huang, Kailin Gao, Lu Yang, Yahai Lu

**Affiliations:** 1 College of Urban and Environmental Sciences, Peking University, No. 5, Yiheyuan Road, Haidian District, Beijing, 100871 China; 2 Key Laboratory of Biology and Genetic Improvement of Oil Crops, Ministry of Agriculture and Rural Affairs/Oil Crops Research Institute of Chinese Academy of Agricultural Sciences, Wuhan, China

**Keywords:** Straw decomposition, Polysaccharides utilization loci, CAZymes gene cluster, Glycoside hydrolase, *Bacteroidota*, *Firmicutes*

## Abstract

**Background:**

Decomposition of plant biomass is vital for carbon cycling in terrestrial ecosystems. In waterlogged soils including paddy fields and natural wetlands, plant biomass degradation generates the largest natural source of global methane emission. However, the intricate process of plant biomass degradation by diverse soil microorganisms remains poorly characterized. Here we report a chemical and metagenomic investigation into the mechanism of straw decomposition in a paddy soil.

**Results:**

The chemical analysis of 16-day soil microcosm incubation revealed that straw decomposition could be divided into two stages based on the dynamics of methane, short chain fatty acids, dissolved organic carbon and monosaccharides. Metagenomic analysis revealed that the relative abundance of glucoside hydrolase (GH) encoding genes for cellulose decomposition increased rapidly during the initial stage (3–7 days), while genes involved in hemicellulose decomposition increased in the later stage (7–16 days). The increase of cellulose GH genes in initial stage was derived mainly from *Firmicutes* while *Bacteroidota* contributed mostly to the later stage increase of hemicellulose GH genes. Flagella assembly genes were prevalent in *Firmicutes* but scarce in *Bacteroidota*. Wood–Ljungdahl pathway (WLP) was present in *Firmicutes* but not detected in *Bacteroidota*. Overall, *Bacteroidota* contained the largest proportion of total GHs and the highest number of carbohydrate active enzymes gene clusters in our paddy soil metagenomes. The strong capacity of the *Bacteroidota* phylum to degrade straw polymers was specifically attributed to *Bacteroidales* and *Chitinophagales* orders, the latter has not been previously recognized.

**Conclusions:**

This study revealed a collaborating sequential contribution of microbial taxa and functional genes in the decomposition of straw residues in a paddy soil. *Firmicutes* with the property of mobility, WLP and cellulose decomposition could be mostly involved in the initial breakdown of straw polymers, while *Bacteroidota* became abundant and possibly responsible for the decomposition of hemicellulosic polymers during the later stage.

**Supplementary Information:**

The online version contains supplementary material available at 10.1186/s40793-023-00533-6.

## Background

Carbon cycling in terrestrial ecosystems is driven by photosynthesis and biomass decomposition. Global carbon storage in terrestrial ecosystems is over 1500 Pg per year, of which over 75–100 Pg carbon is returned to the atmosphere by soil microbial heterotrophic respiration [1]. Rice paddy fields are widely distributed in Asia and provide a staple food for over half of the world populations [2]. The byproduct of plant residues, including rice straw, stubbles, and root debris, amounts to 1.7 billion tons per year, most of which are left in fields and undergo anaerobic decomposition during the growing season of rice plants [3]. This leads to an annual emission of 24–40 Tg methane into the atmosphere [4].

Rice straw residues comprise on average 32–47% cellulose, 19–27% hemicellulose, 5–24% lignin, and a variety of minor organic compounds [5]. These heteropolymers are interwoven to form the stiff indurative structure. How diverse soil microorganisms breakdown straw heteropolymers, however, remains poorly characterized. The previous research revealed the succession of microbial community over the course of rice residue decomposition [6–9]. The bacterial populations during straw decomposition could be separated into three successional groups: an early group consisting mainly of *Clostridium spp.* that increased rapidly after the input of plant residues into soil, a later group including *Bacteroidetes* and *Chlorobi* that increased and gradually surpassed the early group in the later period, and a third group having relatively low abundance without significant fluctuation over time [7]. The early and late successional groups were considered to be the opportunists and generalists taking the r- and K-type life strategies, respectively [7]. The enzymatic assays showed that the activity of b-glucosidase increased rapidly at the beginning while exoglucanase and xylosidase increased gradually during straw decomposition [10]. The successional changes of bacterial populations and enzymatic activities were reflected in the intermediate and end products of organic matter decomposition, in which reducing sugars were detected within hours, followed by rapid accumulation and consumption of hydrogen, and then the production and decomposition of a series of short chain fatty acids (SCFAs) [10]. The reason behind the succession of bacterial populations and hydrolysis enzymatic activities, however, remains unknown.

Carbohydrate active enzymes (CAZymes) [11], comprise glycoside hydrolase (GH), carbohydrate esterase (CE), polysaccharide lyase (PL), glycosyltransferase (GT), auxiliary activity (AA), and accessory noncatalytic carbohydrate-binding module (CBM) [12]. These enzymes often work with other proteins, such as sugar transporters, transcriptional regulators and signal transduction proteins to depolymerize complex carbohydrate substrates [13]. Genes encoding CAZymes and the latter proteins are often arranged into a series of physically linked gene clusters and hence are termed as CAZymes gene clusters (CGCs) [14]. A related term, polysaccharide utilization loci (PUL), is used to represent experimentally characterized CAZymes gene cluster, which sometimes is depicted with the corresponding substrate (e.g., XUL, xylan utilization loci) [15]. PUL initially proposed in *Bacteroidota* includes a hallmark two-component transport system termed SusC/D (archetypal form, Starch utilization system) [16–19]. SusC protein is a unique TonB-dependent trasporter, which in tight association with a substrate-binding lipoprotein SusD, mediates the uptake of oligo- or mono- saccharides via a “pedal bin” mechanism [20, 21]. More recently, PULs were also identified in Gram-positive bacteria like *Firmicutes*, which lack periplasmic space [22]. The genes encoding CAZymes are also arranged into gene clusters but without the *susCD*-like genes [23], which are replaced by genes encoding ATP-binding cassette (ABC) transporters, major facilitator superfamily (MFS) transporters or carbohydrate phosphotransferase system (PTS) [24]. In recent decades, significant progress has been made to mechanistically understand polymers decomposition in gut systems [25–28]. Microbial decomposition of plant polymers in terrestrial ecosystem, however, remains poorly documented.

In the present study, we used microcosm incubations to track the underlying chemical, microbial and function gene changes to gain insight into the mechanism of straw residue decomposition in paddy soil. Our goals are (i) to track straw decomposition process by monitoring intermediate products in the microcosm incubations, (ii) to evaluate the change of key microbial taxa over the course of straw decomposition, and (iii) to unravel catabolic mechanisms by reconstructing environmental genomes of key degraders. We hypothesize that the succession of degrader community and extracellular hydrolytic activity during straw decomposition was due to the collaborated activity of diverse microorganisms in decomposing the complex straw polymers.

## Methods

### Soil incubations

Soil samples were collected from a paddy field located in southern China near to the Anshun city of Guizhou Province (26.05 °N, 105.77 °E) [29]. The chemical properties measured using standard soil testing protocol [30] were: pH 5.3 (with H_2_O), total organic carbon 65.1 g C kg^−1^, total nitrogen 2.42 g N kg^−1^ and total phosphorus 0.311 g P kg^−1^. Soil samples were mixed with autoclaved water at a ratio of 1:5 and 50 ml of homogenized soil slurries were dispensed into 100 ml sterile bottles. Rice straw was dried at 65 °C, cut into ~ 1 mm pieces and applied into soil slurries (1% by mass). The bottles were vigorously shaken by hand to homogenize soil slurries, sealed with black butyl stoppers and aluminum caps, evacuated and flushed with N_2_ for 5 min. Incubation was carried out in the dark at 30 °C under static condition. In total, 15 incubations were prepared. The destructive sampling was conducted at five time points (0 d, 3 d, 7 d, 14 d, 16 d) with triplicate for each time. Soil slurry samples were centrifuged at 10,000×*g*; the resulting supernatants were collected and stored at − 20 °C for chemical analysis; the resulting soil pellets were collected, frozen by liquid nitrogen and stored at − 80 °C for DNA analysis.

### Chemical analysis

Gas sample (200 μl) was collected from the headspace using a gas-tight pressure-lock syringe (Baton Rouge, LA, USA) and analyzed for methane using gas chromatography with a flame ionization detector (Agilent 7890B, USA) [31]. The concentration of methane was calculated following Clapeyron Equation (PV = nRT). Liquid samples stored at − 20 °C were thawed and 1 ml sample was acidified using 5 µl of 18.4 M H_2_SO_4_, filtered through 0.22 μm filters, and analyzed for acetate, propionate, butyrate by High Performance Liquid Chromatography (HPLC, Agilent 1260, USA) with a UV detector at 210 nm [7]. 0.5 ml of liquid sample was subjected to acid hydrolysis using trifluoracetic acid and derivation using 1 ml of 0.5 M PMP-methanol and 0.5 ml of 0.3 M NaOH, then analyzed for monomeric sugars using HPLC (Agilent 1200, USA) with a UV detector at 245 nm [32]. Finally, a fraction of liquid sample was diluted 10 times and analyzed for total dissolved organic matter (DOC) using a total organic carbon analyzer (TOC-L CPN CN2600, SHIMADZU, Japan) [33].

### DNA extraction and metagenomic analysis

Soil DNA was extracted using MP FastDNA SPIN Kit [for soil] (MP Biomedicals, Solon, USA) following the manufacturer’s protocol. DNA sequencing was performed using HiSeqX000 (Illumina) at Allwegene Company (Beijing, China). The image analysis, base calling and error estimation were performed using Illumina Analysis Pipeline v.2.6. In total, 15 DNA samples (in triplicates for each sampling point) were sequenced with an average depth of 20 GB. We obtained a total of 1.19 billion paired-end DNA reads, which were trimmed via Trimmomatic using the default settings [34]. The clean DNA sequences from triplicate samples were assembled individually and then co-assembled via Megahit using the “meta-large” option to obtain 15 metagenomes and 5 co-assemblies, respectively [35]. The open reading frames in the assembled contigs (> 1500 bp) were identified using Prodigal v.2.6.3 [36]. The relative abundance of predicted genes was calculated by mapping raw reads in metagenomes against the identified genes using the software salmon [37]. Co-assembling yielded about 5.1 million scaffolds longer than 500 nucleotides for a total length of 11.7 Gbp. The five co-assemblies were used for binning using an integrated binning software Metawrap, which included three algorithms (Metabat2, Maxbin2 and Concoct) that produced three preliminary binning sets [38]. These preliminary binning sets were used in different combination for bin refinement using the “bin_refinement” module with the hybrid approach settings. The completeness and contamination of the refined bins were evaluated by CheckM v1.1.2 [39]. The relative abundance of bins was calculated by mapping reads onto the contigs in the binned genomes using “quant_bins” function within Metawrap. The taxonomy of the bins was obtained using Genome Taxonomy Database Toolkit (GTDB-tk, v1.3.0) with database GTDB release 95 and default parameters [40].

### Gene analysis for carbohydrate-active enzymes (CAZymes) and the prediction of CAZymes gene clusters (CGCs)

The gene-central and genome-central approaches were used to characterize CAZymes-encoding genes and CGCs across 15 metagenomes. CAZyme-affiliated reads were identified by querying total gene datasets against dbCAN, a database for carbohydrate-active enzyme annotation [41]. In the gene-central approach, taxonomy of GH-encoding genes in 15 metagenomes was determined by aligning GH-encoding gene catalogs against the NCBI NR database using the BLASTp method in the software Diamond v.0.9.14 with settings: “sensitive” model, e-value cutoff of 1e−5 [42, 43]. The relative abundance of GH-encoding genes was calculated by mapping raw metagenomic reads onto the identified GH genes and presented as gene copies per million (CPM) according to the concept of TPM (transcripts per million) used in quantification of transcript expression [37].

In the genome-central approach, the bins with completeness > 90% and contamination < 10% were considered as the high-quality metagenome-assembled genomes (MAGs). These high-quality MAGs were annotated using the software Prokka v1.13 with “metagenome” settings [44] and Prodigal v.2.6.3 [36]. Predicted protein sequences were annotated by a sequence of similarity searches (e-value < 1e−5) based on KEGG database online [45]. The genome phylogeny of the MAGs was constructed using the concatenated multiple sequence alignments of 120 bacterial unique marker genes using “classiy_wf” and “infer” function within GTDB-tk, and visualized by “ggtree” package in R software [40, 46]. CGCs were identified based on the presense of at least one signature gene coding for sugar transporters, signal transduction proteins or transcriptional factors using dbCAN3 [41, 47]. The target substrate prediction of identified CGCs was performed through dbCAN3 based on dbCAN-PUL database and the CAZyme subfamilies using eCAMI tools [41, 47]. Total GHs encoded in individual MAGs were analyzed using the carbohydrate active enzyme database as mentioned above. The secreted signal peptides of GH proteins were predicted using SignalP v.5.0 server online with Gram-negative and Gram-positive models [48]. All identified CGCs in MAGs of *Bacteroidota* and *Firmicutes* are listed in the Additional file 2: Table S1.

The statistical analysis and plotting were done using the R package v4.0.2 [49].

## Results

### Methane, SCFAs and monosaccharides

**Fig. 1:**
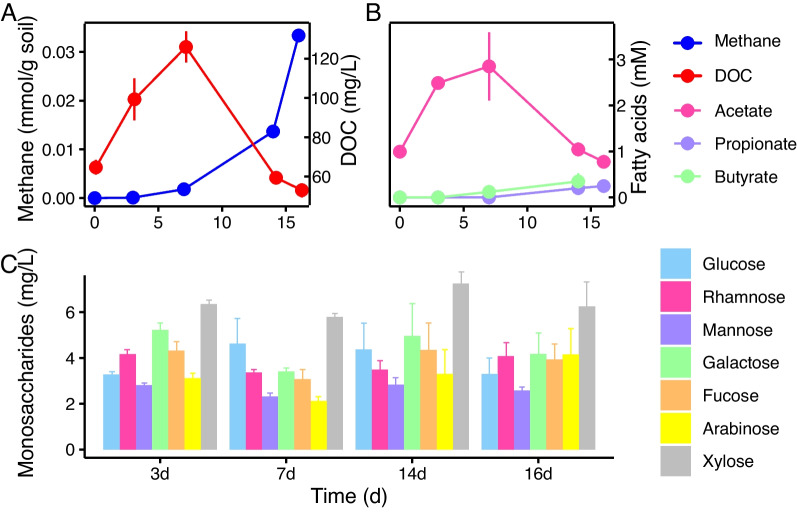
Breakdown products during anaerobic decomposition of rice straw. **A** Concentrations of methane (blue) and dissolved organic carbon (DOC, red). The concentration of methane in the headspace was expressed in millimoles per gram soil (left y-axis) and DOC in milligram C per liter soil slurry (right y-axis). **B** Concentrations (in mM) of acetate, propionate and butyrate in soil slurry. **C** Concentrations (in milligram per liter) of seven monosaccharides in soil slurry, including glucose, rhamnose, mannose, galactose, fucose, arabinose, and xylose. The error bars indicate the standard deviations of three biological replicates

We monitored the dynamics of DOC, methane, SCFAs and monosaccharides during the anaerobic decomposition of rice straw. DOC increased rapidly and peaked (125 mg l^−1^) at 7 d followed by a rapid decrease (Fig. 1A). Methane was detected immediately and increased markedly at 7 d. SCFAs comprised mainly acetate, propionate and butyrate (Fig. 1B). Acetate reached a maximal concentration of 3 mM at 7 d, accounting for approximately 60% of total DOC with the temporal pattern in coincidence with DOC. Propionate and butyrate accumulated slowly to about 0.5 mM at the end of incubation.

Major monosaccharides detected in soil slurries included glucose, mannose, galactose, rhamnose, fucose, arabinose and xylose (Fig. 1C). The total concentration of monosaccharides accounted for ca. 25% to 30% of total DOC. The temporal pattern did not fluctuate significantly and was not correlated to concentrations of DOC or methane. Among seven monosaccharides, xylose showed the highest concentration followed by galactose and glucose. The composition and concentration of monosaccharides reflected the result of their production and consumption over the straw decomposition.

### Dynamics of GH encoding genes

To investigate the temporal dynamic of plant polymer decomposition, we conducted metagenomic analysis of soil slurry samples collected at 0, 3, 7, 14 and 16 days after the incubation. Here we focused on the dynamics of the gene relative abundance coding for glycoside hydrolases, which are known to play the central role in the decomposition of plant polymers [12]. A total of 250 different GH-encoding genes were retrieved across 15 metagenomes (Additional file 1: Fig. S1). The composition and relative abundance exhibited a distinct shift over the decomposition of rice straw (Additional file 1: Fig. S1). In the beginning (0 – 3 d), the genes related to glucanases like GH9, GH5, indicative of the cellulose degradation, were abundant. In the middle stage (7 d), those for cellobiose and glucoside hydrolases like GH48, GH3 increased in together with the gradual increase of genes encoding hemicellulose hydrolases such as mannanase (GH76, GH99) and galactanase (GH135). In the latter stage (7–16 d), the genes indicative of hemicellulose decomposition were substantially enriched, including those encoding galactosidase (GH2, GH27), rhamnohydrolase (GH78, GH106), arabinofuranosidase (GH127, GH146), fucosidase (GH29, GH95) and xylosidase (GH31).

**Fig. 2:**
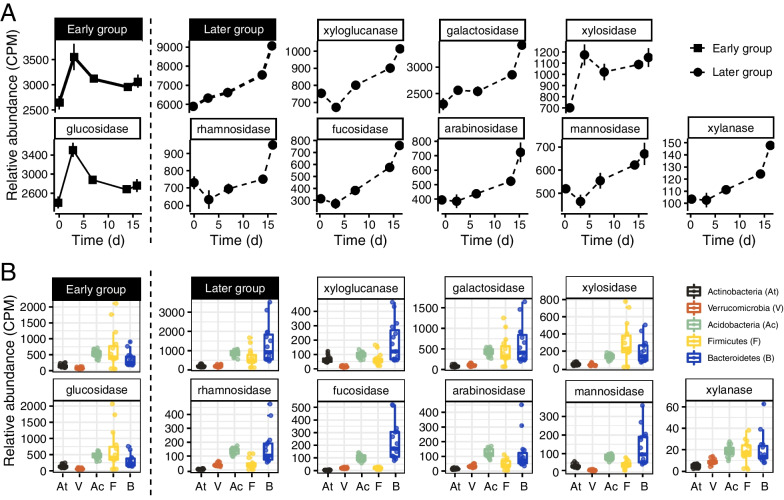
The relative abundances of GH-encoding genes summarized according to catabolic substrates and taxonomic assignment. **A** Changes in the relative abundance of GH-encoding genes over the incubation period show two distinct gene categories: an early group containing mainly the glucosidase-encoding genes that increased rapidly and a later group including genes for galactosidase, xylosidase, xyloglucanase, rhamnosidase, fucosidase, arabinosidase, mannosidase and xylanase that increased gradually over the incubation period. The error bars indicate the standard deviations of three biological replicates. **B** The relative abundances of GH-encoding genes in five main bacterial phyla (At, *Actinobacteria*; V, *Verrucomicrobia*; Ac, *Acidobacteria*; F, *Firmicutes*; B, *Bacteroidota*). The relative abundances of GH-encoding genes (in gene copies per million) were estimated by mapping metagenomic reads against each individual GH-encoding gene

The distinct succession of GHs-encoding genes prompted us to classify GHs into two categories, i.e., an early group and a later group (Fig. 2). The GH encoding genes for cellulose decomposition (mainly glucosidase) were assigned into the early group, of which the gene relative abundance increased rapidly, reaching the maximum at 3 d followed by a gradual decline (Fig. 2A). The later group was indicated by xyloglucanase, xylanase, galactosidase, fucosidase, mannosidase and rhamnosidase, arabinosidase and xylosidase, of which the gene relative abundance increased monotonically over the incubation (Fig. 2A). The genes encoding xyloglucanase, rhamnosidase and mannosidase showed a short drop at 3 d and then increased steadily over the incubation.

The taxonomic classification of GHs-encoding genes revealed that the GHs-encoding genes were mainly derived from five bacterial phyla, *Actinobacteria*, *Acidobacteria*, *Bacteroidota, Firmicutes* and *Verrucomicrobia* (Fig. 2B). *Bacteroidota* contained the most number of GH genes across 15 metagenomes, especially for the later group with xyloglucanase, galactosidase, fucosidase, rhamnosidase and mannosidase (Fig. 2B). *Firmicutes* showed a relatively high contribution to the early group with glucosidase, xylosidase and xylanase. *Acidobacteria* contained a high gene number for rhamnosidase and arabinosidase, whereas *Actinobacteria* and *Verrucomicrobia* contained relatively few GH genes (Fig. 2B).

### Metagenome-assembled genomes (MAGs)

To further elucidate the function and mechanism of different phyla in straw polymer decomposition, we recovered 221 high-quality bacterial MAGs with estimated completeness > 90% and contamination < 10% from our 15 metagenomes (Additional file 3: Table S2). These MAGs belonged to 17 phyla and metagenomic mapping revealed that 6 phyla were present in all sampling points (Additional file 1: Fig. S2): *Acidobacteriota*, *Actinobacteriota*, *Bacteroidota*, *Firmicutes*, *Proteobacteria* and *Verrumicrobiota*. Of all the phyla retrieved, *Bacteroidota* contained the most abundant GH-encoding genes (Additional file 1: Fig. S3).

To examine the dynamics of bacterial populations over the incubation period, we performed 16S rRNA gene amplicon sequencing using soil slurry samples collected at the beginning (0 day) and at 14 days. The results showed that the relative abundances of the *Bacteroidota* and *Firmicutes* phyla increased from 5 to 30% and 10% to 20%, respectively (Additional file 1: Fig. S4). However, the relative abundance of *Proteobacteria* decreased from 45 to 25%. Those of *Actinobacteria*, *Acidobacteria* and *Verrucomicrobia* also decreased to various extents. These results in combination with the distribution of GH-encoding genes in metagenomes (Fig. 2B) suggested that members of *Bacteroidota* and *Firmicutes* played the most important role in straw decomposition. We therefore focused our MAG analysis on these two phyla next.

### MAGs within *Bacteroidota* and *Firmicutes*

**Fig. 3:**
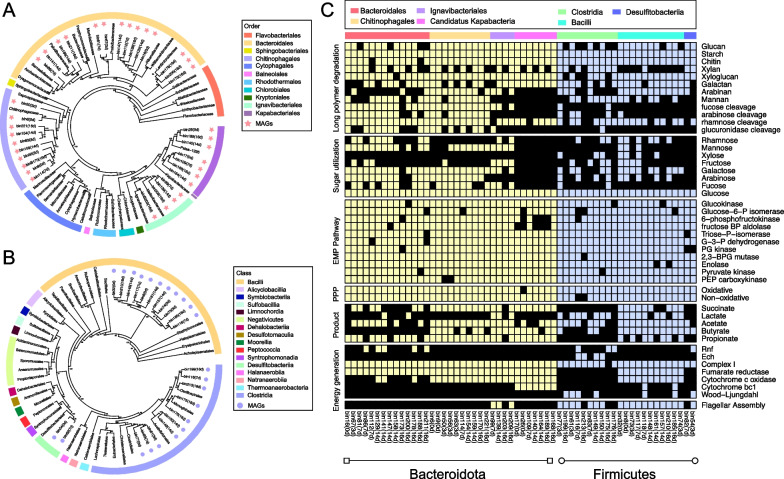
Phylogenetic analysis and metabolic reconstruction of 58 metagenome-assembled genomes (MAGs) within the *Bacteroidota* and *Firmicutes* phyla. **A** Maximum-likelihood genome phylogeny of the 35 retrieved MAGs and 39 reference genomes within *Bacteroidota* based on a group of 120 conserved genes. Different colors in the outer ring indicate different taxonomic orders of *Bacteroidota*. MAGs obtained in this study are indicated by red asterisks, and their incubation time are indicated in parentheses after bin names. **B** Maximum-likelihood genome phylogeny of 23 MAGs and 38 reference genomes within *Firmicutes*. Different colors in the outer ring indicate different taxonomic classes of *Firmicutes*. MAGs obtained in this study are indicated by purple circles. **C** Heatmap reveals the presence or absence of genes or pathways (indicated on the right) related to polysaccharide degradation or metabolite production in the MAGs (indicated at the bottom). The presence of a gene or pathway is denoted by a colored box (*Bacteroidota* in yellow, *Firmicutes* in blue), and black boxes denote the absence of a corresponding gene or pathway in that MAG. At least 60% of the genes in a pathway are required to be present for that pathway to be present. The functional categories (indicated on the left-hand side) include long polymer degradation, sugar utilization, EMP pathway, PPP, intermediate product production and energy generation. MAGs are phylogenetically clustered (shown by different color at the top including: *Bacteroidales* in red; *Ignavibacteriales* in purple; *Chitinophagales* in orange; *Candidatus* Kapabacteria in pink; *Clostridia* in green; *Desulfitobacteriia* in blue; *Bacilli* in cyan). Abbreviations: BP, bisphosphate; BPG, bisphosphoglycerate; G-3-P, glyceraldehyde-3-phosphate; P, phosphate; PG, phosphoglycerate; PPP, pentose phosphate pathway; PEP, phosphoenolpyruvate; Rnf, Ferredoxin:NAD^+^ oxidoreductase; Ech, ech-type hydrogenase

We retrieved 35 and 23 high-quality MAGs taxonomically classified within the phyla *Bacteroidota* and *Firmicutes*, respectively. The *Bacteroidota* MAGs were assigned into three classes (*Bacteroidia*, *Ignavibacteria*, *Candidatus* Kapabacteria) and four orders (*Chitinophagales*, *Bacteroidales*, *Ignavibacteriales*, *Candidatus* Kapabacteria). Specifically, 14 MAGs were distantly related to families *Williamwhitmaniaceae*, *Prolixbacteraceae*, *Paludibacteraceae* and *Lentimicrobiaceae* within the order *Bacteroidales* (Fig. 3A). Nine MAGs formed a cohesive group within the family *Chitinophagaceae*. Eleven MAGs were distantly related to the *Ca*. Kapabacteria and *Melioribacteraceae* or *Ignavibacteriaceae*. The *Firmicutes* MAGs were assigned into three families *Bacilli* (11 MAGs), *Clostridia* (10 MAGs) and *Desulfitobacteriia* (2 MAGs) (Fig. 3B).

Metabolic reconstruction of the above 58 MAGs showed that the capacity to use long chain plant polymers and sugars was prevalent in both *Bacteroidota* and *Firmicutes* (Fig. 3C, the first panel from top). The genes for glucan, starch, chitin and xyloglucan degradation were identified in majority of the MAGs. Apart from the complete glycolysis and pentose phosphate pathway, the capacity of fermentation was found in majority of the MAGs, which could produce various SCFAs including acetate, propionate and butyrate. Most of these MAGs also encoded complex I, fumarate reductase and cytochrome *c* oxidase, indicating the capacity of respiration (Fig. 3C, the second panel from bottom).

Three key differences could be found among the 58 MAGs (Fig. 3C). First, the genes for utilizing the products derived from hemicellulose decomposition including mannose, galactose, fucose, arabinose, and glucuronide were more often encoded in the MAGs of *Bacteroidales* and *Chitinophagales* than in the other MAGs (Fig. 3C, the second panel from top). Xylose utilization, however, was encoded in a few *Firmicutes* MAGs but absent in the *Bacteroidota* MAGs. Intriguingly, despite the capacity of plant polymer breakdown widely encoded in *Ca.* Kapabacteria, these organisms did not have genes for utilizing monosaccharides except glucose. Second, the Wood–Ljungdahl pathway was encoded in some *Firmicutes* but not in *Bacteroidota*. Third, flagellar assembly proteins were encoded in most of the *Firmicutes* MAGs but rarely in *Bacteroidota* (Fig. 3C, the first panel from bottom).

**Fig. 4:**
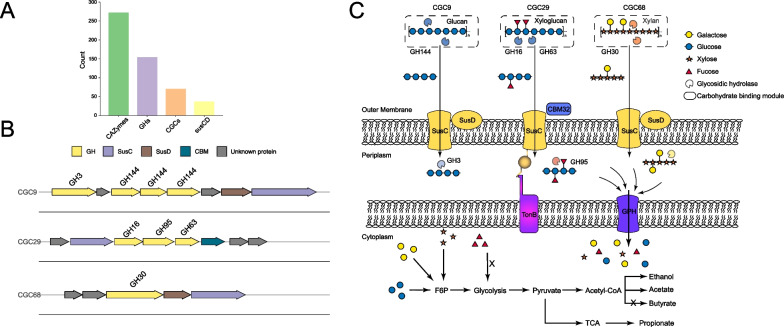
Hypothetical model of the degradation and utilization of plant heteropolymers by *Chitinophagales* inferred from a reconstructed genome (MAG_bin63). **A** The number of encoded CAZymes, GHs, CGCs and *susCD* copies. **B** Gene cluster diagrams of three representative CGCs. **C**, Hypothetical model depicting the deconstruction of glucan, xyloglucan and xylan by CAZymes encoded in three CGCs. Polymers are depolymerized by secreted GHs; the oligosaccharide products are transported into periplasm using outer membrane SusCD lipoprotein complexes and hydrolyzed by periplasmic GHs; the periplasmic products are internalized and finally fermented to various products like acetate, propionate and ethanol. The protein cellular locations are based on predicted N-terminus secretion signal peptides. Monosaccharides are represented using the standard Consortium of Functional Glycomics symbols for glycans (galactose, yellow circle; glucose, blue circle; xylose, orange asterisk; fucose, red triangle) [66]. Abbreviations: CGC, CAZymes gene cluster; GH, glycoside hydrolase; *susCD*, starch utilization system; F6P, fructose 6-phosphate; GPH, glycoside-pentoside-hexuronide transporter; TCA, tricarboxylic acid cycle

We took a representative MAG (bin63) within *Chitinophagaceae* to depict the possible process of straw polymer degradation (Fig. 4). The MAG bin63 contained in total 70 CGCs, 37 *susCD*, and 272 CAZyme encoding genes including 154 GH genes of which 60% bearing the secretion signal (Additional file 2: Table S1, Fig. 4A). In the hypothetical model, straw polymers such as glucans, xyloglucans and xylans are depolymerized into oligosaccharides by the extracellular GHs encoded in three representative CGCs (Fig. 4B, C). The produced oligosaccharides are transported into periplasm via the SusCD complex and degraded into di- and mono- saccharides by glucosidase (GH3), fucosidase (GH95) encoded in corresponding CGCs. The di- and mono- saccharides are imported into cytoplasm via the transporters like glycoside-pentoside-hexuronide transporter (GPH transporter) for further metabolism and finally acetate and propionate are produced through the fermentation of monosaccharides (Fig. 4C).

### Diversity of predicted CAZymes gene clusters (CGCs) within *Bacteroidota* and *Firmicutes*

**Fig. 5:**
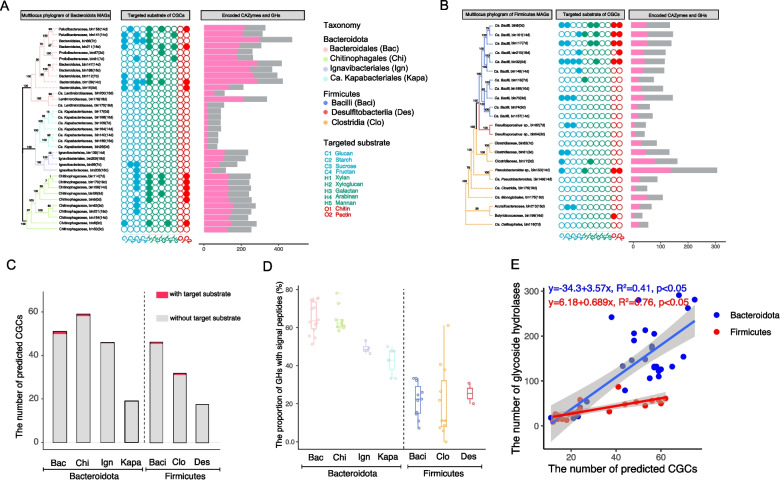
Properties of CGCs encoded in the *Bacteroidota* and *Firmicutes* MAGs. **A**, **B** The maximum likelihood phylogenetic tree reconstructed based on 120 concatenated marker genes (left panel), targeted substrate prediction of CGCs (middle panel) and the total numbers of CAZymes and GH proteins (right panel) encoded in the individual MAGs of *Bacteroidota* (**A**) and *Firmicutes* (**B**), respectively. Full description of CGCs and detected proteins are given in Table S1. Targeted substrates are indicated by closed circles and colored by substrate identity. Different lineage clades in phylogenetic trees are colored by taxonomic order (*Bacteroidota*) or class (*Firmicutes*). **C** The average numbers of predicted CGCs with and without targeted substrates in *Bacteroidota* and *Firmicutes*. Notably, very limited proportions of predicted CGCs had the matched substrates in dbCAN-PUL database. **D** The proportion of GHs with signal peptides in *Bacteroidota* and *Firmicutes*. **E** Spearman rank correlations (one-tailed) between the number of predicted CGCs and the number of glycoside hydrolases (blue, *Bacteroidota*; red, *Firmicutes*). The solid colored line indicates the fitted one-tailed Spearman correlation (R^2^), and the grey bands indicate the corresponding 95% confidence intervals for the curve. The regression equations and correlation coefficients are shown if statistically significant (*p* < 0.05). Abbreviations: CGC, CAZymes gene cluster; MAG, metagenomic assembled genome; GH, glycoside hydrolase

CGC is a gene cluster arrangement encoding CAZymes for polysaccharides metabolism. We analyzed the CGC properties in 58 MAGs affiliated to *Bacteroidota* and *Firmicutes* (Fig. 5). The representative structure and composition of CGCs were illustrated in Additional file 1: Fig. S5. The presence of *susCD* was considered a canonical hallmark in the well characterized PULs within *Bacteroidota* [17]. The *susCD* genes were present in most CGCs of *Bacteroidales* and *Chitinophagales*, but scarcely detected in the CGCs of *Ignavibacteriales* and none in *Ca.* Kapabacteria (Additional file 1: Fig. S5A). The genes encoding ABC transporter, PTS and MFS, which are known for sugar transport [24] were present in the *Firmicutes* CGCs (Additional file 1: Fig. S5B). The cellulosome-encoding genes were occasionally detected.

We analyzed the total numbers of predicted CGCs and CAZyme genes in the 58 MAGs. *Bacteroidota* encoded an average of 50 CGCs per MAG, which was two-fold than the average of 25 CGCs per MAG in *Firmicutes* (Fig. 5). *Chitinophagales* of the *Bacteroidota* phylum encoded the highest CGC number (on average 58 CGCs per MAG), while *Ca*. Kapabacteria and *Desulfitobacteriia* solely contained an average of 20 CGCs per MAG (Fig. 5C). In accordance with the CGC number, total CAZymes and GHs encoded in the *Bacteroidota* MAGs were much more abundant than the *Firmicutes* MAGs (Fig. 5A, B). The MAGs of the orders *Bacteroidales* and *Chitinophagales* encoded the most abundant and diverse CAZymes and GHs (on average 280 CAZymes and 170 GHs per MAG), whereas the *Firmicutes* MAGs contained on average 111 CAZymes and 43 GHs per MAG.

We further predicted the targeted carbohydrate substrates of identified CGCs using dbCAN3 [41]. Intriguingly, only 5% of the identified CGCs in *Bacteroidota* and *Firmicutes* had the matched substrate identities (Fig. 5C). Some *Bacteroidota* like two *Ignavibacteriales* MAGs (bin139 and bin209) encoded about 55 CGCs but none of them had the matched substrates. Likewise, two novel *Bacteroidales* species represented by bin147 and bin188 encoded around 250 GHs and 40 CGCs but without a matched glycan substrate (Fig. 5A) in the dbCAN-PUL database. Nevertheless, the substrate-matched CGCs of *Bacteroidota* and *Firmicutes* displayed a different substrate spectrum. The *Bacteroidota* CGCs tend to be more involved in the degradation of hemicelluloses including xylan, galactan and mannan, while the *Firmicutes* CGCs displayed a possible preference for xyloglucan and chitin utilization (Fig. 5B).

The secretion of CAZymes is crucial for plant polymer decomposition. We analyzed GH proteins encoded in 58 MAGs for secretion signal peptides at the protein N-terminus (Fig. 5D) [50]. Approximately half of the GH proteins in *Bacteroidales*, *Chitinophagales* and *Ignavibacteriales* contained secretion signals and hence were possibly secreted. *Ca.* Kapabacteria contained not only fewer numbers of CGCs but also fewer GH proteins carrying secretion signals. About 20–30% of the GH proteins in *Firmicutes* contained signal peptides, which was far lower than that in *Bacteroidota* (Fig. 5D). In addition, correlation analysis revealed a significant linear relationship between the number of predicted CGCs and the number of encoded GHs in both *Bacteroidota* and *Firmicutes* (Fig. 5E). The slope of linear correlation indicated that an individual *Firmicutes* CGC encoded about 1 GH, while an average of 3–4 GHs were encoded in the *Bacteroidota* CGCs, possibly illustrating the higher metabolic diversity of predicted CGCs in *Bacteroidota* than those in *Firmicutes*.

## Discussion

Our study demonstrates that the decomposition of rice straw in paddy soil is dependent on the collaborating function of diverse microorganisms. During the initial stage of straw decomposition, we found a rapid increase of glucosidase-encoding genes that indicate cellulose cleavage (Fig. 2). This initial cleavage of cellulose could disintegrate the straw polymer structure and increase accessibility to microorganisms. Consequently, GHs for hemicellulose decomposition increased substantially and became abundant during the later stage of straw decomposition (Fig. 2). Taxonomic analysis revealed that *Bacteroidota* and *Firmicutes* played important but different roles in the straw decomposition: *Firmicutes* were likely to be more important during the early stage of cellulose cleavage, whereas *Bacteroidota* were likely to be more involved in the hemicellulose decomposition during the later stage.

Flagella were encoded in most of the *Firmicutes* MAGs (Fig. 3C) and could provide these microorganisms better access to straw polymer substrates [51–53]. Furthermore, a few members of *Firmicutes* encoded Wood–Ljungdahl pathway, which could support efficient usage of glucose resulting from cellulose decomposition to produce acetate. This is supported by our chemical measurements, in which we identified a rapid production of acetate during the early stage and a subsequent sharp increase of methane at 7 day, likely as a result of aceticlastic methanogenesis (Fig. 1). Thereafter, *Bacteroidota* gradually became abundant in the later stage of straw decomposition. The increased decomposition of hemicellulose was reflected by the slight increments of monosaccharides, including arabinose, fucose and rhamnose, from 7 day onwards (Fig. 1C). The slow but steadily accumulation of propionate and butyrate during the late stage (Fig. 1B) were probably also due to the activity of *Bacteroidota*, given that the genes for these products are prevalently encoded in their reconstructed genomes (Fig. 3C). It has been documented that the decomposition and nutrient uptake of dietary glucans in human intestine are controlled largely by the population ratio of *Firmicutes* relative to *Bacteroidota* (hereafter F/B ratio) in human gut microbiome [54]. A high F/B ratio corresponded to a faster utilization of glycans that possibly led to a high obesity risk [55]. We estimated the F/B ratio during the course of incubation and found the temporal pattern of F/B ratio (Additional file 1: Fig. S6) was in coincidance with the dynamics of DOC and acetate (Fig. 1). The high F/B value possibly indicated the dominance of initial cellulose clevage by *Firmicutes* and over time F/B value decreased implying the substrate transition from cellulosic polysaccharides to hemicellulosic polysaccharides.

The *Bacteroidota* community in paddy soil comprised at least four orders: *Bacteroidales*, *Chitinophagales*, *Ignavibacteriales* and *Ca.* Kapabacteria (Fig. 3A). We evaluated their potential in plant polymer decomposition based on the numbers of CGCs, total GHs and the proportion of secreted GHs encoded in their MAGs. Intriguingly, the enrichment of CGCs and total GH proteins occurred only in the orders *Bacteroidales* and *Chitinophagales* (Fig. 5A). *Chitinophagales* are rarely detected in gut [56, 57], and to our knowledge, have not been documented in plant polymer decomposition in paddy soils. Currently, only a small number of *Chitinophaga* species have been isolated and characterized from soil environments [58–60] and most of these cultured species are aerobes that respire plant sugar polymers with oxygen [61–63]. Here we obtained 9 *Bacteroidota* MAGs within the family *Chitinophagaceae* and the predicted CGCs revealed these *Chitinophagaceae* had a broad substrate utilization potential such as for galactan and arabinan (Fig. 5A). These results indicate that *Chitinophagales* encode remarkable capacity to decompose straw polymer in anoxic paddy soil.

Apart from *Bacteroidales* and *Chitinophagales*, 11 of 35 *Bacteroidota* MAGs are clustered with *Ignavibacteriales* and *Ca.* Kapabacteria. Three of the four *Ignavibacteriales* MAGs contain flagella assembly genes that are absent in other *Bacteroidota* MAGs (Fig. 3C, Additional file 1: Fig. S4A). Furthermore, although *Ca.* Kapabacteria MAGs encoded most of extracellular enzymes for the heteroglycan deconstruction, the genes for utilizing monosaccharides except glucose were rarely detected (Fig. 3C). These genomic characteristics indicated that albeit the phylogenetic affiliation within *Bacteroidota*, the catabolic machineries of *Ignavibacteriales* and *Ca.* Kapabacteria for plant polymer utilization are distinct from *Bacteroidales* and *Chitinophagales*.

In addition, *Acidobacteriota*, *Actinobacteria* and *Verrucomicrobiota* also contributed to the CAZymes pool in paddy soil. Particularly, the proportion of GH genes in *Acidobacteria* is comparable to that in *Firmicutes* (Fig. 2B). A recent study showed that *Actinobacteria* played an important role in straw decomposition in paddy field soil in situ [64]. However, our study showed that the relative abundances of *Acidobacteria* and *Actinobacteria* decreased over 14 days incubation (Additional file 1: Fig. S3), suggesting that these organisms might be less competitive in utilizing straw polymers in comparison with *Bacteroidota* and *Firmicutes*.

PULs are known to play critical roles in carbohydrate utilizations in *Bacteroidota* [22, 65]. However, only a few PULs have been characterized with the biological sources mainly from gut bacteria while other habitats especially soils have been scarcely documented [62, 63]. In the present study, we found majority of predicted CGCs in *Bacteroidota* and *Firmicutes* MAGs did not have a predicted substrate based on current dbCAN-PUL database [14]. Given the complexity of plant biomass heteropolymers and high diversity of soil degraders, characterization of soil microbiome-origin CGCs could provide a deeper understanding on the key microbial players and genes involved in soil organic matter decomposition.

## Conclusions

Collectively, our study provides chemical and genomic evidence for the capabilities of *Bacteroidota* and *Firmicutes* in rice straw anaerobic decomposition and describes links between temporal change of these microorganisms and plant residues decomposition. While the contributions of *Firmicutes* and *Bacteroidota* to plant residues decomposition in human intestine and animal rumen have been well documented in the past, our findings fill a knowledge gap for the complex microbial decomposition of plant residues in anoxic paddy soil environments.

### Supplementary Information

**Additional file 1: Fig. S1.** Succession of 250 GH-encoding genes during anaerobic decomposition of rice straw. The five columns represent 5 sampling points (0 d, 3 d, 7 d, 14 d, 16 d) indicated at the bottom of the figure. Values are the mean of three replicates. The relative abundance of GH-encoding genes was presented as gene copies per million (CPM), which was normalized based on the Z-score method. The colored key indicates the Z-score values. GHs-encoding genes were hierarchically clustered based on their average Euclidean distance values. **Fig. S2.** The succession of different metagenomic-assembled genomes (MAGs) during anaerobic decomposition of rice straw. Different phylum MAGs were indicated by colored circle and the difference of relative abundance is expressed in scales. **Fig. S3.** The average number of encoded glycoside hydrolases in six dominant phyla including *Bacteroidota*, *Verrucomicrobiota*, *Acidobacteriota*, *Firmicutes*, *Proteobacteria* and *Actinobacteriota*. **Fig. S4.** The relative abundance of bacterial community during anaerobic decomposition of rice straw. Top ten phyla are shown for indication of total bacterial communities with the rest classified into the “Others” groups. **Fig. S5.** Representative CAZymes gene clusters (CGCs) identified in *Bacteroidota* (**A**) and *Firmicutes* (**B**). The reference genomes include *Bacteroides xylanisolvens*, *Hydrotalea flava*, *Ignavibacterium album* and *Kapabacteria bacterium*, *Bacillus subtilis*, *Clostridium cellulovorans*, and *Desulfitobacterium hafniense*. Abbreviations: CBM, carbohydrate binding module; GT, glycoside transferase; CE, carbohydrate esterase; GH, glycoside hydrolase; PL, polysaccharide lyase; PTS, carbohydrate phosphotransferase system; MFS, major facilitator superfamily. **Fig. S6.** The abundance ratio of *Firmicutes* relative to *Bacteroidota* (F/B). The relative abundances of *Firmicutes* and *Bacteroidota* were estimated by mapping metagenomic reads against metagenomic assembled genomes (MAGs) of *Firmicutes* and *Bacteroidota*, respectively.

**Additional file 2: Table S1.** Description of predicted CGCs within *Bacteroidota* and *Firmicutes* phyla.

**Additional file 3: Table S2.** Detailed information of metagenomic-assembled genomes

## Data Availability

All sequencing data generated in this study were deposited in National Center for Biotechnology Information (NCBI) under the BioProject ID PRJNA944798. Raw metagenomic reads have been deposited in the Sequence Read Archive (SRA) under the accession numbers SAMN33761923-SAMN33761937. The 221 high-quality MAGs have been deposited in the same project.

## Abbreviations

AA: Auxiliary activity enzyme
CAZymes: Carbohydrate active enzymes
CBM: Carbohydrate-binding module
CE: Carbohydrate esterase
CGC: CAZymes gene cluster
DOC: Dissolved organic carbon
GH: Glycoside hydrolase
GT: Glycoside transferase
Pg: Petagram
PUL: Polysaccharides utilization loci
SCFA: Short chain fatty acid
Tg: Teragram
