# Supplementary figures and images for: Successional action of Bacteroidota and Firmicutes in decomposing straw polymers in a paddy soil
Source: Environ Microbiome. 2023 Oct 14;18:76. doi: 10.1186/s40793-023-00533-6 (PMC10576277; doi:10.1186/s40793-023-00533-6)

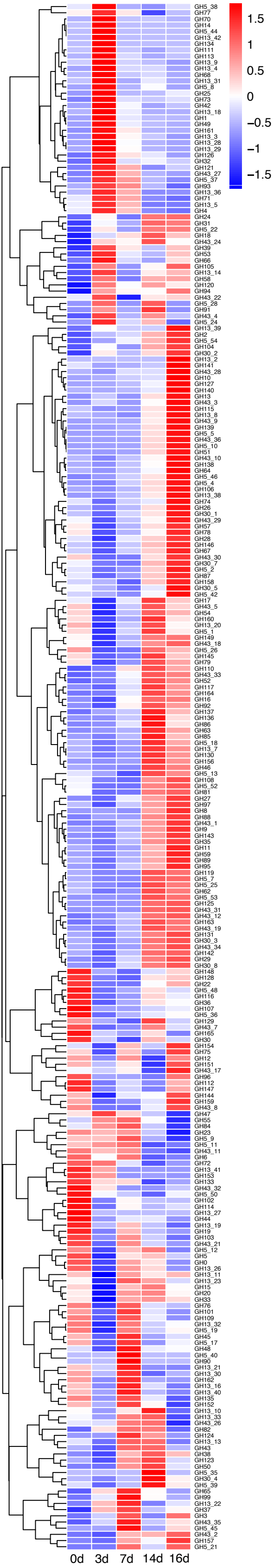

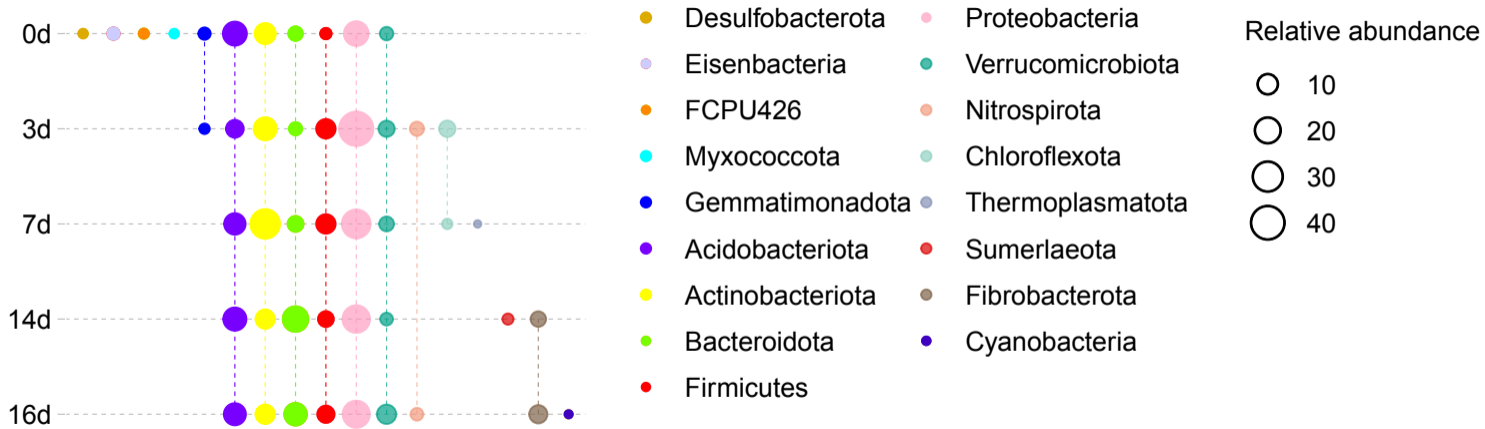

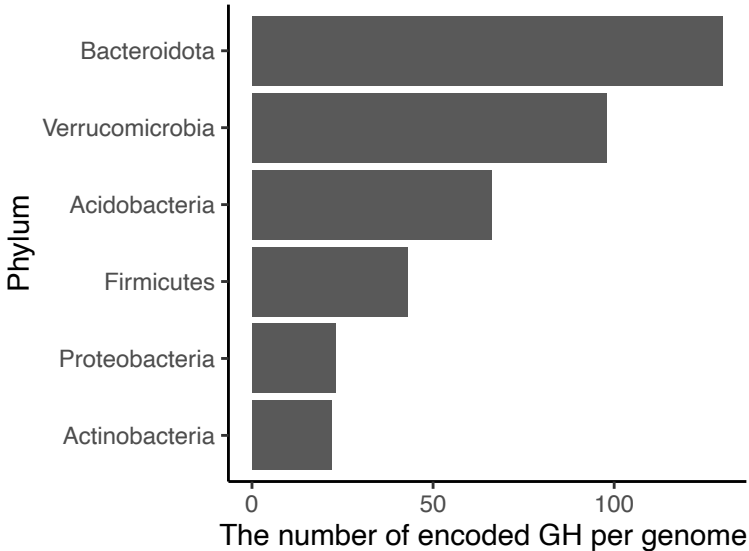

A

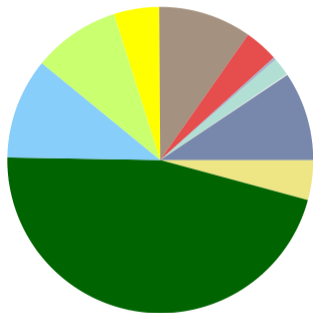

0d

B

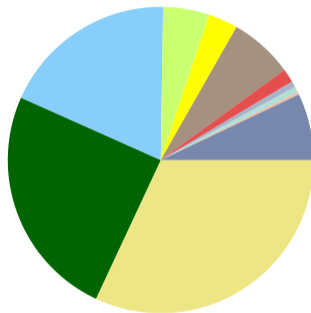

14d

Phylum

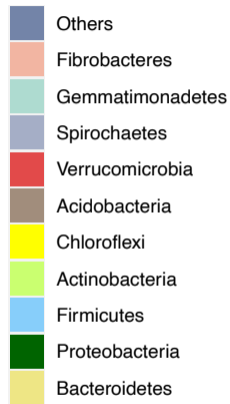

# A

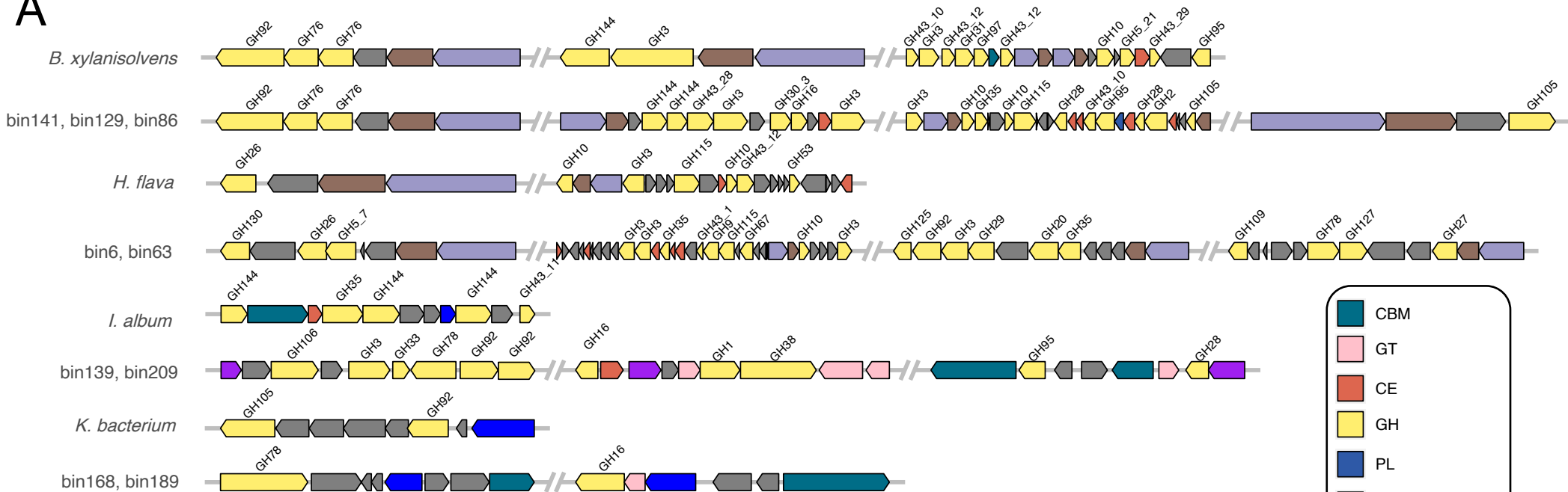

# B

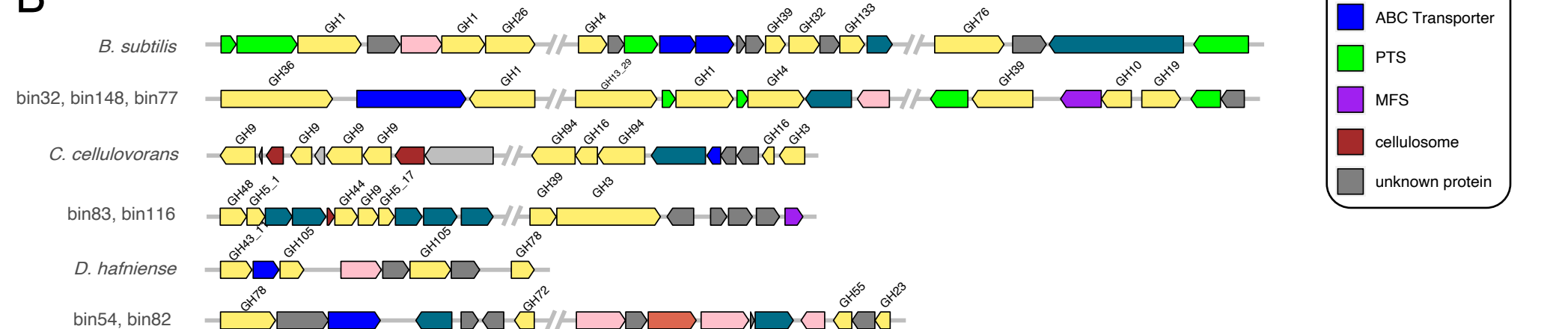

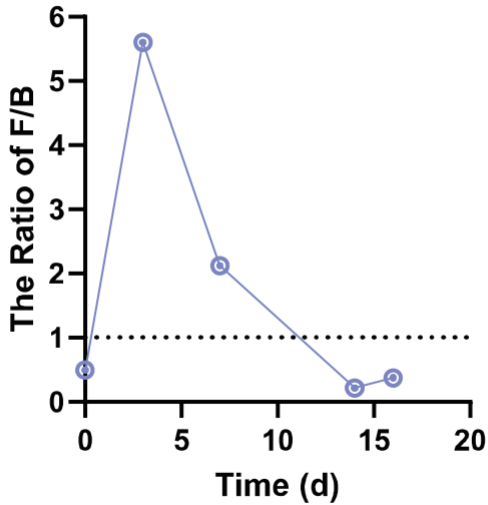

Supplement: Supplementary file 1 — Additional file 1: Fig. S1. Succession of 250 GH-encoding genes during anaerobic decomposition of rice straw. The five columns represent 5 sampling points (0 d, 3 d, 7 d, 14 d, 16 d) indicated at the bottom of the figure. Values are the mean of three replicates. The relative abundance of GH-encoding genes was presented as gene copies per million (CPM), which was normalized based on the Z-score method. The colored key indicates the Z-score values. GHs-encoding genes were hierarchically clustered based on their average Euclidean distance values. Fig. S2. The succession of different metagenomic-assembled genomes (MAGs) during anaerobic decomposition of rice straw. Different phylum MAGs were indicated by colored circle and the difference of relative abundance is expressed in scales. Fig. S3. The average number of encoded glycoside hydrolases in six dominant phyla including Bacteroidota, Verrucomicrobiota, Acidobacteriota, Firmicutes, Proteobacteria and Actinobacteriota. Fig. S4. The relative abundance of bacterial community during anaerobic decomposition of rice straw. Top ten phyla are shown for indication of total bacterial communities with the rest classified into the “Others” groups. Fig. S5. Representative CAZymes gene clusters (CGCs) identified in Bacteroidota (A) and Firmicutes (B). The reference genomes include Bacteroides xylanisolvens, Hydrotalea flava, Ignavibacterium album and Kapabacteria bacterium, Bacillus subtilis, Clostridium cellulovorans, and Desulfitobacterium hafniense. Abbreviations: CBM, carbohydrate binding module; GT, glycoside transferase; CE, carbohydrate esterase; GH, glycoside hydrolase; PL, polysaccharide lyase; PTS, carbohydrate phosphotransferase system; MFS, major facilitator superfamily. Fig. S6. The abundance ratio of Firmicutes relative to Bacteroidota (F/B). The relative abundances of Firmicutes and Bacteroidota were estimated by mapping metagenomic reads against metagenomic assembled genomes (MAGs) of Firmicutes and Bacteroidota, re [file 40793_2023_533_MOESM1_ESM.pdf]
